# Screening for Chlamydia trachomatis in Egyptian women with unexplained infertility, comparing real-time PCR techniques to standard serology tests: case control study: Chlamydia trachomatis and unexplained infertility

**DOI:** 10.1186/s12905-015-0202-5

**Published:** 2015-06-02

**Authors:** Rana M. A. Abdella, Hatem I. Abdelmoaty, Rasha H. Elsherif, Ahmed Mahmoud Sayed, Nadine Alaa Sherif, Hisham M. Gouda, Ahmed El Lithy, Maged Almohamady, Mostafa Abdelbar, Ahmed Naguib Hosni, Ahmed Magdy, Youssef MA

**Affiliations:** Department of obstetrics & gynecology, Faculty of Medicine, Cairo University, Cairo, Egypt; Department of clinical pathology, gynecology, Faculty of Medicine, Cairo University, Cairo, Egypt; Egyptian International Fertility IVF-ET center, 16 Elhassan Ben Ali, Nast City, Cairo Egypt

**Keywords:** Unexplained infertility, *Chlamydia* trachomatis, PCR, Antisperm antibodies

## Abstract

**Background:**

To study the prevalence of *Chlamydia* infection in women with primary and secondary unexplained infertility using ELISA technique for antibody detection and real time, fully automated PCR for antigen detection and to explore its association with circulating antisperm antibodies (ASA).

**Methods:**

A total of 50 women with unexplained infertility enrolled in this case control study and a control group of 44 infertile women with a known cause of infertility. Endocervical specimens were collected for *Chlamydia* antigen detection using PCR and serum samples for antibodies detection. Circulating anti-sperm antibodies were detected using sperm antibody Latex Agglutination tests.

**Results:**

The overall prevalence of *Chlamydial* infection in unexplained infertility cases as detected by both ELISA and PCR was 40 % (20/50). The prevalence of current *Chlamydial* genital infection as detected by real-time PCR was only 6.0 % (3/50); two of which were also IgM positive. Prevalence of ASA was 6.0 % (3/50); all were sero-negative for anti-*C.trachomatis* IgM and were PCR negative.

**Conclusion:**

The incidence of *Chlamydial* infection in Egyptian patients with unexplained infertility is relatively high. In the setting of fertility investigations; screening for anti. *C.trachomatis* antibodies using ELISA, and treatment of positive cases should be considered. The presence of circulating ASA does not correlate with the presence of old or current *Chlamydia* infection in women with unexplained infertility.

## Background

Unexplained infertility (UI) is a diagnosis that is reached when all the standard investigations such as tests for ovulation, tubal patency tests and semen analysis are normal [1, 2]. The average incidence of unexplained infertility varies from 0 % to 37 % [3]. *Chlamydia trachomatis* is the most common sexually transmitted bacterial pathogen [4]. It is estimated that over 90 million cases occur annually worldwide attesting to its public health importance. More than two-thirds of these cases occur in the developing world where diagnostic and treatment facilities are almost absent [5].

*C.trachomatis* of the lower genital tract is predominantly asymptomatic in men and women. Between one half and two thirds of such infections in women remain undetected and hence untreated, resulting in serious long-term sequelae, such as ectopic pregnancy and tubal infertility [6, 7]. Consequently, screening is necessary to identify and treat this infection to help reduce duration of infectivity, transmissibility and long term sequelae [8–10].

Culture testing for *C. trachomatis* has been the reference standard against which all other tests have been compared. As culture methods are difficult to standardize, technically demanding and expensive, other tests have been developed [11]. Nucleic acid amplification techniques (NAAT) are currently being used to diagnose chlamydial infections. NAATs can be used with noninvasively collected specimens, such as first-void urine samples (FVU) from men or women and self- or clinician-collected vaginal swabs leading to increased acceptance of *C. trachomatis* screening programmes among asymptomatic persons [12, 13].

Reliable estimates of prevalence data for genital Chlamydia infection using sensitive and specific techniques like nucleic acid amplification tests are lacking in developing countries as Egypt. Few available reports describe an increased incidence of genital Chlamydial infection, especially among symptomatic Egyptian women [14]. There is an ongoing debate as to whether or not screening of all infertile women and treatment of those found to be infected is desirable or cost effective. Hence, the present pilot study was designed to determine the prevalence of *C.trachomatis* infection (past and current) in Egyptian females with unexplained infertility using ELISA and real-time fully automated PCR (Real-TM PCR) and evaluate its association with circulating anti-sperm antibodies.

## Methods

From September 2013 to February 2014, we enrolled 50 infertile couples with unexplained infertility seeking conception who attended the infertility outpatient clinic and the Assisted Conception Unit at Cairo university hospital and the Egyptian International fertility IVF – ET center (EIFC-IVF), Cairo, Egypt. The study participants presented with primary or secondary unexplained infertility for more than 2 years and were recruited from a total of 215 infertile women examined. The study protocol was approved by the Scientific and Ethical Committee of the Obstetrics and Gynecology Department of Cairo University.

The diagnosis of unexplained infertility was based on: documentation of ovulation by trans-vaginal ultrasonography and normal mid-luteal serum progesterone levels, documentation of normal uterine cavity by hysteroscopy, bilateral tubal patency by hysterosalpingography/ laparoscopy as indicated and normal semen parameters by WHO criteria [15]. The exclusion criteria were: history of pelvic surgery including cesarean section, history of pelvic inflammatory disease and clinical features suggestive of pelvic pathology such as, endometriosis, uterine fibroids and ovarian cysts,previous use of intrauterine contraceptive device especially in cases with secondary infertility.

Later, we included a control group of 44 women presenting with primary or secondary infertility either due to male factor only with no identifiable female factor; women with the diagnosis of PCO; menstrual irregularities and/or uterine fibroids. Those with evident tubal factor of infertility as diagnosed by hysterosalpingography and laparoscopy or premature ovarian failure were excluded from the control group as well as the previously mentioned exclusion criteria for the cases. The need to include a control group emerged when we were faced by the unexpectedly high prevalence of Chlamydia trachomatis infection among the first group of unexplained infertility cases and due to the lack of previous studies in Egypt clearly addressing this issue. A question was raised as to whether this high prevalence is specific to cases of unexplained infertility only or is it also a common finding in infertile women without an evident tubal factor of infertility or dense pelvic adhesions or endometriosis which are usually conditions difficult to treat. An informed, written consent was obtained from all participants prior to enrollment and was written in the Arabic language.

Endocervical swabs were collected by the attending physician. The swab was inserted into the endocervical canal for a distance about 1-1.5 cm until most of the swab tip was inside the external os. In nulliparous women, swabs were inserted as far as could be reached. Swabs were left in place for about 5–10 s while applying pressure or scraping to obtain more cells from the endocervix and were subsequently expressed in 5 ml of phosphate buffer saline (PBS) transport medium for Chlamydial antigen detection. The phosphate buffer saline (PBS) was prepared as described by Dulbecco and VogtinSambrook et al. [16]. Detection of the *C.trachomatis DNA* in the collected cervical specimens was done using the Real-TM PCR by amplification. Real-TM PCR(TaqMantechnology, ABI 7500, AppliedBiosystems) was coupled with an automated DNA extraction method (QIAGEN). Extraction of DNA was performed using the QI Amp DNA mini kit (50) (QIAGEN GmbH, Hilden, Germany Cat. No. 51304). Primers and probe *for* Real-TM PCR*assay* were selected from sequences of the cryptic plasmid (GenBank accession nos M19487, Y00505, J03321, X06707 and X07547) of five different *C. trachomatis* strains (serotypes A, B, D, L1 and L2, respectively), and designed using Primer Express software (Applied Biosystems)). A forward primer Ctr_F (59-CATGAAAACTCGTTCCGAAATAGAA-39),a reverse primer Ctr_R (59TCAGAGCTTTACCTAACAACGCATA-39)(which amplify a 71 bp DNA segment of *C. trachomatis*) and aminorgroove binder probe labeled with 59FAM (6-carboxyfluorescein) Ctr_P (59TCGCATGCAAGATATCGA-39) were selected. The melting temperature (Tm) of the probe was chosen to be 10–11 °C higher than that of the corresponding primers, in order to ensure probe hybridization during primer extension. The primers were prepared by Eurogentec and the probe by Applied Biosystems. The reactions were performed in a final volume of 20 μl, including 0.2 μM each primer, 0.1 μM Ctr_P probe, 10 μl 26 TaqManUniversal Master Mix (Applied Biosystems) and 5 μl DNA sample. Cycling conditions were 2 min at 50 °C, 10 min at 95 °C, followed by 45 cycles of 15 s at 95 °C and 1 min at 60 °C. Amplification and PCR product detection were performed with the ABI Prism 7900 Sequence Detection system (Applied Biosystems) [16].

Interpretation of the real-time TaqMan PCR was done as follow*;* During amplification, the reporter dye (FAM) was measured against the passive reference dye (ROX) signal to normalize fluorescence fluctuations not related to PCR amplification and that may occur with increasing cycle numbers. A positive result was determined by identifying the threshold cycle (Ct), i.e. the cycle number at which normalized reporter dye emission was above the background noise (corresponding to ten times the standard deviation of the mean baseline emission calculated for PCR cycles 3–15). If the fluorescent signal did not increase within 45 cycles, the sample was considered negative [17].

Serodiagnosis for Anti-*C.trachomatis IgG and IgM:* About 3-5 ml of peripheral venous blood samples were collected for all patients and were allowed to stand to clot. The serum was then separated by centrifugation.

The serum was examined using the ELISA Technique (enzyme-linked immune-sorbent assay) for detection of the anti-C.trachomatis IgG and IgM. The ratios of IgM and IgG were also calculated. Detection of *C. trachomatis* specific IgG and IgM by ELISA: Circulating anti *C. trachomatis* IgG antibodies were detected in serum by ELISA (enzyme-linked immunosorbent assay) using RIDASCREEN®*Chlamydia trachomatis*, KGM2901, R-Biopharm Darmstadt, Germany) which provides materials for the semi qualitatitive determination of IgG and IgM-classes antibodies to *C. trachomatis* in serum. Specimen collection, preparation, assay procedure, calculation and interpretation of results were done according to the manufacturer's instructions.

The average absorbance is calculated for the cut-off control and the sample index (SI) is obtained by dividing the absorbance for the sample by the calculated average value of IgG & IgM as follows; negative, SI <0.9, equivocal, SI 0.9 to 1.1 and positive, SI >1.1. Blood for determination of circulating Antisperm Antibody (ASA, IgG class) was drawn. ASA in serum was assayed by Sperm Ab Latex Agglutination tests (BIOSERV Diagnostics, R-Biopharm, Germany) which were done according to the manufacturer's instructions. Results were communicated with the patient and treatment in form of doxycycline 100 mg twice daily for seven days was offered to the patient and her partner on diagnosis.

Data were statistically described in terms of mean ± standard deviation (± SD), median and range, or frequencies (number of cases) and percentages when appropriate. Comparison of numerical variables between the study groups was done using Mann Whitney *U* test for independent samples. For comparing categorical data, Chi square (*χ*^2^) test was performed. Exact test was used instead when the expected frequency is less than 5. Correlation between various variables was done using Pearson moment correlation equation for linear relation in normally distributed variables and Spearman rank correlation equation for non-normal variables. *p* values less than 0.05 was considered statistically significant. All statistical calculations were done using computer program SPSS (Statistical Package for the Social Science; SPSS Inc., Chicago, IL, USA) release 15 for Microsoft Windows (2006).

## Results

Ninety-four women consented to participate in this study; a first group of 50 cases with unexplained infertility and a control group of 44 women with a known cause of infertility. Both groups were comparable regarding the age and BMI where the mean age (± S.D) of the cases group was 30.78 ± 5.81 years (range from 19 to 40 years) and for the control group was 30.68 ± 6.11 years. The mean infertility duration was 5 ± 3.19 years for the cases while it was 3.93 ± 1.62 years for the controls with a statistically significant difference. Thirty-six out of 50 cases (72 %) had primary unexplained infertility and 14 out of 50 cases (28 %) had the secondary type (Table 1). The mean gravidity was 1.67 ± 1.04. Thirteen out of 50 cases (26 %) had a previous failed IUI (46 % of which were IgG + ve) and 6.0 % had a failed ICSI trial (all were seronegative for anti- *C.trachomatis* IgG and IgM).

**Table 1:** Comparison of the clinical and demographic characteristics between the unexplained infertility cases and the controls

Variable	Cases (n = 50)	Controls (n = 44)	*p*-values
Age (yrs)*	30.78 (19-41)	30.68 (19-40)	0.893
Body mass index (kg/m^2^)*	30.26 ± 2.21	31.07 ± 1.69	0.211
Duration of Infertility (yrs)*	3.93 (2.5-10)	5.0 (2-16)	0.041
< *5 years*	26 (52 %)	31 (70.5 %)	0.068
*≥5 years*	24 (48 %)	13 (29.5 %)	
Type of infertility			
*Primary*	36 (72 %)	35 (79.5 %)	0.396
*Secondary*	14 (28 %)	9 (20.5 %)	
Previous parity	9 (18 %)	6 (13.6 %)	0.564
Previous abortion	10 (20 %)	6 (13.6 %)	0.413
Previous ICSI/IUI	17 (34 %)	9 (20.5 %)	0.308
Anti C.*trachomatis* IgM + ve	3/50 (6 %)	0/44 (0 %)	0.099
Anti C.*trachomatis* IgG + ve	18 (36 %)	8 (18.2 %)	0.054
Ratio of IgM*	0.37 ± 0.26	0.59 ± 0.24	*<* 0.05
Ratio of IgG*	1.26 ± 0.99	0.87 ± 0.63	0.03
PCR positive	3/50 (6 %)	0/44 (0 %)	0.09
Antisperm Abs positive	3/50 (6 %)	1/44 (2.3 %)	0.372

*Values are expressed in terms of mean ± S.D. Other values are expressed in terms of frequency and %. S stands for significant (p < 0.05); NS stands for non-significant

**Fig. 1:**
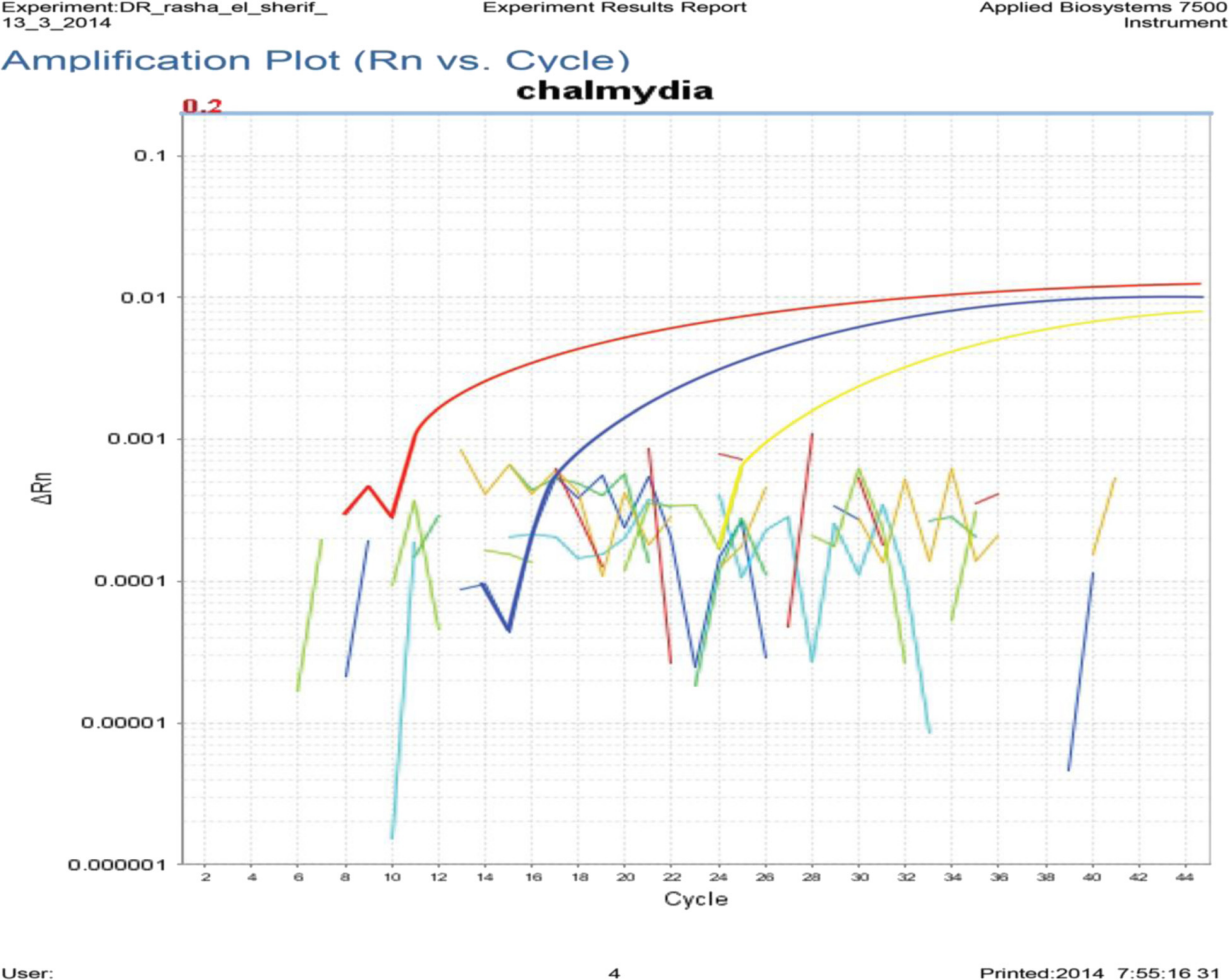
Graphical representation of real-time PCR data. Rn is the fluorescence of the reporter dye divided by the fluorescence of a passive reference dye; i.e., Rn is the reporter signal normalized to the fluorescence signal of ROX™. The figure demonstrate an amplification plot that show the variation of log (ΔRn) with PCR cycle number and as seen in the figure there are three positive cases that are above the threshold cycle (ct)

The overall prevalence of *Chlamydial* (past and current) infection as detected by both ELISA and Real-TM PCR in the unexplained infertility group was 40 % (20/50) while the prevalence in the control group was much lower of 18.2 % (all of them being only IgG sero-positive indicating past Chlamydial infection) where none of the controls were positive for IgM Abs or PCR positive. Real-TM PCR identified current *Chlamydial* infection in 3 (6 %) of the case group (Fig. 1), two of which were IgM positive and all 3 cases were of the secondary infertility type and this finding was statistically significant (p = 0.019). The prevalence of anti-*C.trachomatis* antibodies (ACTA) (IgG and IgM Abs) in the unexplained infertility population was 19/50 (38 %). Only three (6.0 %) cases were seropositive for IgM Abs of which 2 were also IgG and PCR positive (Table 2). The mean ratio for anti -*Chlamydia trachomatis* antibodies (ACTA) of IgG type was 1.26 ± 0.99 (range: 0.4 - 4.3) while the mean ratio for the IgM type was 0.37 ± 0.26 (range: 0- 1.4) where a ratio <0.9 was considered negative and > 1.1 considered positive. Eight cases had IgG ratios >2 indicating higher levels of circulating anti-*C.trachomatis* IgG. Equivocal results for IgG (with ratio = 0.9-1.1) were found in three cases (6 %) (Table 2).

**Table 2:** Prevalence of Anti-*C.trachomatis* antibodies (IgG and IgM) in the serum of unexplained infertility cases in relation to the type and duration of infertility

Anti-C.trachomatis antibodies	Type of infertility		Total (n = 50)	Duration of infertility		
	*1ry (n = 36)*	*2ry (n = 14)*		<*5 years (n = 26)*	*>5 years (n = 24)*	
*Data presented with frequency (%)*						
*IgM + ve*	1.0 (2.8 %)	2.0 (14.3 %)	3.0 (6 %)	2.0 (7.7 %)	1.0 (2.9 %)	
*IgM-ve*	35 (97.2 %)	12 (85.7 %)	47 (94 %)	24 (92.3 %)	34 (97.1 %)	
*IgG + ve*	14 (38.9 %)	4.0 (28.6 %)	18 (36 %)	9.0 (34.6 %)	9.0 (25.7 %)	
*IgG –ve*	19 (52.7 %)	10 (71.4 %)	29 (58 %)	15 (57.6 %)	24 (68.6 %)	
*Equivocal IgG*	2.0 (5.6 %)	1.0 (7.1 %)	3.0 (6 %)	2.0 (7.7 %)	2.0 (5.7 %)	
The mean ratios for anti-C.*trachomatis* IgG and IgM using the ELISA technique.						
*Data presented with mean ± S.D*			*P*			*p*
Ratio of IgG^*^	1.29 ± 1.0	1.19 ± 1.0	0.63	1.22 ± 0.9	1.31 ± 1.0	0.52
Ratio of IgM^*^	0.37 ± 0.2	0.42 ± 0.3	0.52	0.38 ± 0.2	0.37 ± 0.2	0.42

*Values are expressed in terms of mean ± S.D.; *p*-value, ratios >1.1 considered positive and <0.9 considered negative

Regarding the unexplained group, anti- *C.trachomatis* IgM was found in the serum of 2.0/14 (14.3 %) of the secondary infertile women and in 1.0/36 (2.8 %) of the primary type and this difference was statistically non-significant (p = 0.186). The presence of IgM Abs did not seem to be statistically related to the duration of infertility (Table 2).

The majority of cases positive for IgG: 14/18 (77.8 %) were of the primary type of unexplained infertility. This represents 38.9 % (14/36) of the primary type within the study group. While 22.2 % of the seropositive IgG were of the secondary type representing 28.6 % of total number of secondary infertility cases, however this relation was found to be statistically non-significant (*p*-value = 0.744). The presence of current *Chlamydial* infection did not seem to be related to the duration of unexplained infertility as shown in (Table 3). Real-time PCR was confirmatory of current Chlamydial infection in 6 % of women with unexplained infertility, all 3 positive cases were of the secondary infertility type and this finding was statistically significant (p = 0.019).

**Table 3:** Prevalence of current genital C.trachomatis infection by PCR in the unexplained group in relation to type and duration using the PCR technique

	Type of infertility			Infertility duration		
	1ry	2ry	*P*	<5 years	≥5 years	*p*
Ct PCR + ve (n = 3)	0.0 (0 %)	3 .0 (21 %)	0.019	1.0 (4.0 %)	2.0 (8.0 %)	0.602
Ct PCR-ve (n = 47)	36 (100 %)	11 (79 %)		25 (96 %)	22 (92 %)

Anti-sperm antibodies (ASA) were detected in three (6 %) of the unexplained cases. Only 1 case with positive ASA was also seropositive for IgG Abs (ratio = 1.2) indicating past *Chlamydial* infection and 1 case had an equivocal result for IgG Abs (ratio = 0.9). None of the cases with current *Chlamydia* infection had ASA (Table 4). In the control group,only 1/44 was positive for antisperm antibodies (2.3 %) which was seronegative for IgG and IgM Abs; leading to a statistically non-significant difference between case and control group(p = 0.372) (Table 1).

**Table 4:** Relationship between ASA and current genital C. trachomatis infection by PCR and anti-C. trachomatis IgG antibodies

	Chlamydial Ag by PCR		Anti-C. trachomatis IgG		equivocal
	+ve	-ve	+ve	-ve	
ASA					
+ve	0.0	3.0 (6 %)	1.0 (6.0 %)	1.0 (3.0 %)	1.0 (33 %)
-ve	3.0	47 (94 %)	17 (94 %)	28 (97 %)	2.0 (67 %)
Total	3.0	50	18	29	3.0
ASA: Antisperm antibodies					

## Discussion

*Chlamydia* screening programs aims to reduce the morbidity from upper genital tract complications and the prevalence of the disease by controlling its transmission [18]. Furthermore, pre-existing asymptomatic infections can be disseminated when infertile patients undergoes uterine instrumentation for further evaluation and treatment of their fertility problem. Hence, the Royal College of Obstetricians and Gynecologists recommends that all patients undergoing uterine instrumentation should be screened for *Chlamydia* spp. or should receive prophylactic antibiotics [19].

This study was conducted on a randomly selected group of women with unexplained infertility seeking ICSI/IVF, aiming to detect the prevalence of Chlamydial infection using ELISA for detection of anti-C.trachomatis IgG and IgM and Real-TM PCR for Chlamydial antigen detection. We then included a control group of infertile women with a known cause of infertility (excluding tubal factor or pelvic pathology), when we were faced with the high prevalence in the first selected group.

The prevalence of *Chlamydia* genital infection and other sexually transmitted diseases in Egypt and most Arab countries is not exactly known, reflecting the limited specific diagnosis and treatment programmes. Various small studies from different countries report different level of *C. trachomatis*; United Arab Emirates (2.6 %) [20], Jordan (3.9 %) [21], Qatar (5.3 %) [22], and Saudi Arabia (15 %) [23]. This variation in prevalence is related to age of the participants, population studied, as well as the different methodologies used. Alarmingly, in our study population of unexplained infertility cases, the overall prevalence of *C. trachomatis* was 40 %. However,a lower prevalence of 18.2 % was detected in the control group, where none of the cases showed evidence of recent chlamydial infection in terms of IgM seropositivity or PCR., Al-Ramahi et al. studied the *Chlamydia* prevalence through PCR only, on 152 infertile Jordanian women, and showed a prevalence of current *Chlamydia* infection similar to ours of 3.9 % [21]. However, when considering only the patients with unexplained infertility in their studied group (66/152), the prevalence varied to 3 % where only 2/66 were PCR + ve. This slightly lower ratio of detection may be related to the technique of PCR where they used the conventional type. The authors further explained that the low prevalence may be attributed to social and cultural conservative nature of the Jordanian society regarding free sexual relations [21].

A meta-analysis performed in 2002 concluded that DNA amplification techniques performed best for both urine and swabs in low prevalence populations [24]. Two systematic reviews found that the use of an endocervical swab gave greater sensitivity than first void urine with sPCR and BD Probetec [25, 26]. In the current study we elected to use Real-TM PCR analysis of endocervical swabs since it is easier to perform, faster, more accurate. It is also performed in a closed system, and as such is less prone to contamination than conventional PCR. Furthermore, when coupled with an automated DNA extraction method, Real-TM PCR made it possible to process a large number of specimens in an almost fully automated procedure, with a low requirement for laboratory technician time [27].

There is a close correlation between the presence of anti-*Chlamydia* antibodies in females and tubal factor infertility. Antibody testing for anti-*Chlamydia* antibodies has been found to be associated with tubal factor of infertility. Increasingly high titers of anti-*Chlamydial* IgG and anti- CHSP60 antibodies have been correlated with increasing severity of tubal damage when evaluated using HSG or laparoscopy [28]. The sensitivity and specificity of *Chlarnydia* IgG antibody test (CAT) are comparable to that of HSG alone. In addition CAT is less cost prohibitive than PCR and has less risks than either HSG or laparoscopy [29, 30].

In spite of high prevalence of *Chlamydial* seropostivity (38 %) among the unexplained group in the present study, relationship to infertility is not clear since patients do not show any signs or symptoms suggestive of pelvic inflammatory disease and have normal tubal patency on investigations. However, occult damage of the tubal cilia or mucosa could not be ruled out. Additionally, infection could have been limited to the lower genital tract only. Moreover, mainly persistent *C. trachomatis* infections, rather than cleared infections, are associated with an increased risk of tubal pathology [28, 31, 32].

In addition, past infection with C.*trachomatis* did not seem to leave a deleterious impact on the control group (accounting for an 18 % prevalence), where none of the women gave history of PID or showed evident tubal/pelvic pathology or affection on diagnostic investigations performed. However still, it can't be determined whether this old infection could add to the already existing cause of infertility in such women later on, or could be implicated perhaps through subtle endometritis or subclinical salpingitis, which necessitates close follow up of such infertile women after possible treatment of the currently apparent cause of infertility.

It has been hypothesized that Chlamydial infection may impair fertility through the generation of anti-sperm antibodies (ASA) as a part of the inflammatory response, which in turn may impair sperm migration and/or block sperm-ovum interaction [33–35]. Also, it has been proposed that there may be an existing cross-reactivity between antigens of *Chlamydia trachomatis* and spermatozoa [36–38]. In the current study no relationship was found between past or current *C. trachomatis* infection and ASA in women with unexplained infertility. This indicates that chlamydial infections have a low probability of inducing circulating anti-sperm antibodies in women. Similar results were reached in the study of Siam and Hefzy [39].

The limitations of the data surveyed in this study include its restriction to the tertiary referral infertility unit, the time frame of 6 month due to the expense of PCR kits, and to a specific risk group.

## Conclusion

We documented higher than expected *C trachomatis* prevalence reflecting lack of STI-specific programmes in Egypt. Review of these data led to a change of the pre IVF workup policy in our unit, with the introduction of *Chlamydia* serological screening and antibiotic treatment of positive cases. We hope to use the results of this study to help design and complete larger clinical trials involving the use of endometrial curettings for the detection of *Chlamydial* antigen by DNA amplification. This may lead to improved identification and characterization of this subgroup of women whose infertility is currently unexplained.

## Abbreviations

ASA: Antisperm antibodies
UI: Unexplained infertility
ICSI/IVF: Intracytoplasmic sperm injection/In vitro fertilization
PCR: Polymerase chain reaction
ELISA: Enzyme-linked immunosorbent assay
NAAT: Nucleic acid amplification techniques
PBS: Phosphate buffer saline
CAT: Chlamydia antibody test
HSG: Hysterosalpingography
